# Landslide Susceptibility Mapping with Integrated SBAS-InSAR Technique: A Case Study of Dongchuan District, Yunnan (China)

**DOI:** 10.3390/s22155587

**Published:** 2022-07-26

**Authors:** Zhifu Zhu, Shu Gan, Xiping Yuan, Jianming Zhang

**Affiliations:** 1 Faculty of Land and Resources Engineering, Kunming University of Science and Technology, Kunming 650093, China; 20202101047@stu.kust.edu.cn (Z.Z.); gskmust@163.com (X.Y.); 2021128525168@stu.kust.com (J.Z.); 2 Application Engineering Research Center of Plateau and Mountainous Spatial Information Surveying and Mapping Technology, Yunnan Universities, Kunming 650093, China; 3 Key Laboratory of Cloud Data Processing and Application of Mountain Scenic Spot in Yunnan Universities, West Yunnan University of Applied Science, Dali 671006, China

**Keywords:** Dongchuan district, integration, SBAS-InSAR, landslide susceptibility, correction

## Abstract

Landslide susceptibility maps (LSM) are often used by government departments to carry out land use management and planning, which supports decision makers in urban and infrastructure planning. The accuracy of conventional landslide susceptibility maps is often affected by classification errors. Consequently, they become less reliable, which makes it difficult to meet the needs of decision-makers. Therefore, it is proposed in this paper to reduce classification errors and improve LSM reliability by integrating the Small Baseline Subsets-Interferometric Synthetic Aperture Radar (SBAS-InSAR) technique and LSM. By using the logistic regression model (LR) and the support vector machine model (SVM), experiments were conducted to generate LSM in the Dongchuan district. It was classified into five classes: very high susceptibility, high susceptibility, medium susceptibility, low susceptibility, and very low susceptibility. Then, the surface deformation rate of the Dongchuan area was obtained through the ascending and descending orbit sentinel-1A data from January 2018 to January 2021. To correct the classification errors, the SBAS-InSAR technique was integrated into LSM under the optimal model by constructing the contingency matrix. Finally, the LSMs obtained before and after correction were compared. Moreover, the correction results were validated and analyzed by combining remote sensing images, InSAR deformation results, and field surveys. According to the research results, the susceptibility class of 66,094 classification error cells (59.48 km^2^) was significantly improved in the LSM after the integration of the SBAS-InSAR correction. The enhanced susceptibility classes and the spectral characteristics of remote sensing images are highly consistent with the trends of InSAR cumulative deformation and the results of field investigation. It is suggested that integrating SBAS-InSAR and LSM is effective in correcting classification errors and further improving the reliability of LSM for landslide prediction. The LSM obtained by using this method plays an important role in guiding local government departments on disaster prevention and mitigation, which is conducive to eliminating the risk of landslides.

## 1. Introduction

As one of the most catastrophic geological disasters worldwide, landslides are causing substantial casualties and economic losses each year [1,2]. Especially in southwest China, there are various geological hazards frequently posed by the complexity of geological formations, stratigraphy, and lithology, which makes it prone to landslides. Therefore, it is practically significant to reduce the damage caused by disasters effectively. In most cases, it is costly to reinforce all unstable slopes, which is not the best option for local governments and those affected. Monitoring and forecasting are regarded as the most cost-effective measures to reduce landslide risk, which are universally applicable. As an important tool for landslide prevention management [3], landslide susceptibility maps offer a primary option for preventing landslide risk as they are applicable to predict the relative spatial probability of landslide occurrence. It carries the basic data for landslide risk evaluation, thus providing a reference for disaster prevention and mitigation. Moreover, it provides the final data as guidance on land use planning [4].

Currently, the approaches to conventional landslide susceptibility mapping methods can be divided into two categories in general: knowledge-driven and data-driven. The former includes the fuzzy logic model (FL) [5,6], the analytic hierarchy process (AHP) [7,8], and the interval pairwise comparison matrix (IPCM) [9]. They are adopted mainly to identify the contributors to landslides based on expert experience, determine the weight of human factors and natural factors, and build regional landslide susceptibility maps. The latter is purposed to generate landslide susceptibility maps by statistically analyzing the contributors to landslides and establishing quantitative prediction models, such as mathematical and statistical models and machine learning models. With the rapid development of computer technology, remote sensing (RS), and geographic information systems (GIS), it has been made easier to collect landslide spatial data, and various data-based methods of data-driven vulnerability modeling are widely applied [10], such as the logistic regression (LR) [11,12], random forest (RF) [13,14], support vector machine (SVM) [15,16], and artificial neural network (ANN) [17,18]. They all have shown high prediction accuracy and efficiency in the practice of susceptibility mapping. In [19], a section along the northern Himalayas in India was taken as an example to map the LSM of the region through LR, as verified in the field. It was found that 72% of the landslide areas were concentrated in the high and very high susceptibility zones according to the susceptibility results, which indicates the feasibility of LR in landslide susceptibility mapping. In [20], the LSM of the Loess Plateau region in Shanxi was obtained by using three models of informativeness, the weight of evidence, and logistic regression, respectively. A test was conducted on the predictive ability of the models with test samples, revealing that the accuracy rate exceeded 80% for the logistic regression model. Tjis evidences the high reliability of the logistic regression model in the LSM. In [21], with a specific region of Serbia in the Frushkagera Mountains selected for the study, the LSM constructed by SVM and AHP was compared in terms of the K-index, the area under the curve (AUC) of the receiver operating characteristic (ROC) curve, and the false alarm rate in the stable ground. In addition, SVM was found to outperform AHP. In [22], the LSM of Hanyuan County, China was mapped by using the generalized additive model (GAM), support vector machine (SVM), and adaptive neuro-fuzzy inference system (ANGIS) combined with frequency ratios, to validate their accuracy against ROC. As suggested by the results, all three models performed well in making predictions, with the support vector machine model achieving the highest prediction rate of 0.875. As demonstrated by all the above studies, both LR and SVM have a strong generalization ability and achieve high accuracy. However, the accuracy of LSM is closely related to historical landslide data, causal variables, and the exact modeling method. Due to the incompleteness of manually-investigated landslide data and the static nature of variables, LSM may encounter many classification error problems under conventional models. For example, the area prone to landslides is mistakenly classified as a stable area, or the stable area is mistakenly classified as a landslide area. These classification errors that contradict reality will increase the uncertainty of LSM to a certain extent, which will significantly reduce the economic value of land and even endanger the safety of life and properties. Nevertheless, there is still little attention paid in previous studies to developing effective solutions to reducing misclassification in LSM.

In recent years, emerging remote sensing technology has provided new means for landslide investigation. InSAR technology has become mainstream with the advantages of high accuracy detection of surface deformation and wide area identification of potential landslides [23]. At present, InSAR technology has been proven to have high feasibility and reliability for landslide identification, which can perform long-time, high-precision overall deformation monitoring of slopes [24,25,26]. Zhang et al. [27] used SBAS-InSAR to investigate landslides in the Zhouqu area of China from December 2007 to August 2010, and identified 11 active mudflows, 19 active landslides with deformation rates exceeding 100 mm/year, 20 new unstable slopes, and demonstrated the excellent performance of InSAR technology in landslide investigation in combination with field survey results. Zhou et al. [28] obtained the deformation results of the Muyubao landslide by the SBAS-InSAR method and verified the reliability of SBAS-InSAR with global positioning system monitoring data to reveal the deformation characteristics of the landslide.

Based on the advantages of InSAR technology in landslide identification, we propose a method in this paper to integrate SBAS-InSAR and LSM while ensuring the accuracy of the susceptibility model and introducing dynamic surface deformation information into LSM to correct the classification error, which compensates for deficiencies of previous LSM studies’ failure to improve classification errors which makes it difficult to provide a reliable reference for decision-makers. In this experiment, the Dongchuan district, which is faced with severe geological hazards, was chosen to verify the proposed method, so as to provide a valuable reference for disaster prevention and mitigation as well as land use planning.

## 2. Study Area

**Figure 1:**
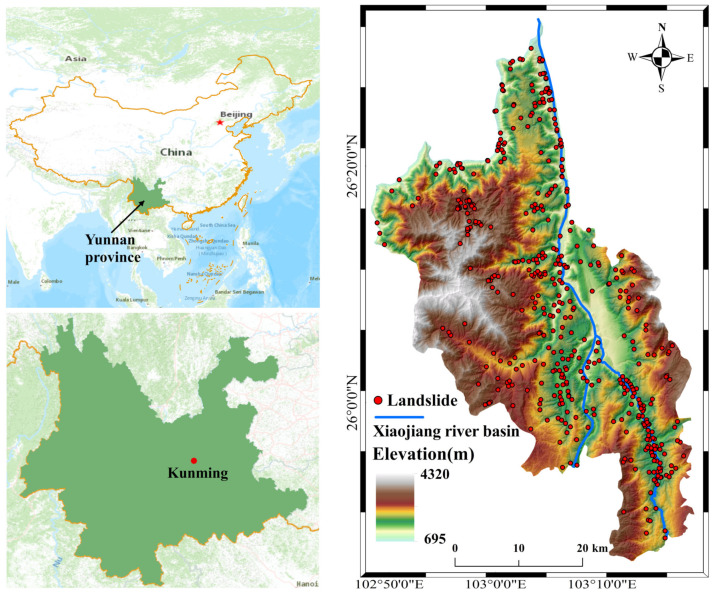
Geographical location and landslide distribution in the study area.

The Dongchuan district falls within the jurisdiction of Kunming city, Yunnan province. It is located in the northeast of Yunnan province and the northernmost part of Kunming city, bordering Huize county to the east, Xundian county to the south, Luquan county to the west, and Huidong county and Huili county in Sichuan province across the Jinsha River to the north [29]. Geographically, it is positioned between 102°47′–103°18′ E and 25°57′–26°32′ N (Figure 1). The lithology covers three major rock types: metamorphic rocks, magmatic rocks, and sedimentary rocks. The shallow metamorphic rocks of the Kunyang group emerged first, Mesozoic red dust was prevalent all over the southwest, and quaternary concentrated mainly in the intermountain basin and river valley area [30]. The process of rock formation has long been affected by tectonic movements and various climatic factors, which leads to broken rock bodies, the development of joints, and the soft structural surfaces that affect their engineering and geological properties.

The Xiaojiang river basin runs through the study area from south to north, on both sides of which the high mountain valley is bounded by the Xiaojiang river. The valley is depressed and conforms to a ‘V’-shaped distribution, which makes it a typical deep-cut high mountain valley. The area is significantly affected by the economically developed Xiaojiang Fracture Zone, and the surface rock layer is broken and poorly stabilized. At the same time, the rainy season in the study area lasts from May to September, with an average annual rainfall of about 1000.5 mm. The localized single-point heavy rainfall in summer creates favorable conditions for the development of geological hazards due to abundant rainfall and strong erosion [31]. This results in the frequent occurrence of various geological disasters such as landslides and debris flows. According to the relevant statistics, between the 1960s and the 1990s, large-scale landslides and mudslides became disastrous on 33 occasions, causing 211 deaths, 90 injuries, and destroying 2133.3 hectares of farmland. The total economic loss exceeded 150 million yuan [32], which makes it a major constraint on regional economic development and social stability.

## 3. Materials and Methods

### 3.1. Data and Variables

In this experiment, there were 458 landslides identified through field surveys and historical data collection up until January 2021. These historical landslides involve three types of geological hazards: landslides, collapses, and debris flows. They are mainly distributed along the high mountain valley areas on both sides of the Xiaojiang River basin (Figure 1). By means of GIS, the landslide area was transformed into 96,966 landslide points, with an equal number of non-landslide points randomly generated to comprise the sample set for the model input. In total, 80% of the samples were used for model training, and the remaining 20% were used to test the model on training accuracy.

In the current practice of landslide susceptibility modeling, there are no unified criteria applied to the selection of causative variables, but the fundamental principle is always to ensure that the variables are operational, measurable, and non-redundant [33]. According to previous studies, the selection of variables is not judged by number, because too many variables contribute nothing to improving the accuracy of LSM. On the contrary, it reduces the accuracy of LSM by introducing noise [34]. Therefore, a total of 11 influencing factors for land use type were adopted as the main indicators of LSM in this paper: lithology, distance to a river, profile curvature, plane curvature, NDVI index, distance to road, aspect, slope, elevation, and fluctuation. Meanwhile, the objective accuracy of the causative variables was ensured during the geological survey. Besides this, they were replaced by X1 to X11, respectively, for the subsequent processing. The sources of experimental data are as follows: (1) 250 m spatial resolution lithology map with 1:250,000 road and water system vector data from the Geoscience Service Platform (China); (2) 30 m spatial resolution land use data collected from the Resource and Environment Science and Data Center of the Chinese Academy of Sciences (China); (3) DEM with 30 m resolution from NASA SRTM (USA) for slope, aspect, plane curvature, profile curvature, elevation, and fluctuation; (4) vegetation cover index extracted from geospatial data cloud Landsat8 (USA) data with 30 m resolution; (5) ascending and descending sentinel-1A data from European Space Agency (ESA) for the deformation rate of the study area. The specific parameters are shown in Table 1.

**Figure 2:**
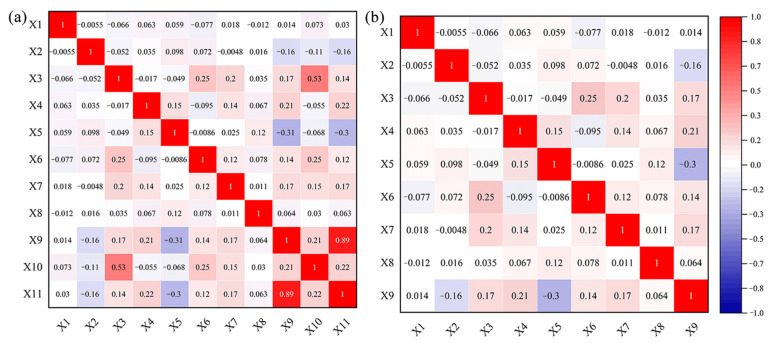
Correlation coefficient matrix. (**a**) is the correlation matrix of 11 variables, and (**b**) is the correlation matrix after excluding elevation (X10) and undulation (X11), where X1 to X11 represents land use type, lithology, distance to a river, profile curvature, plane curvature, NDVI index, distance to road, aspect, slope, elevation, and fluctuation, respectively.

In addition, it is essential to ensure that the selected susceptibility indicators can meet the requirements of the model. To achieve this purpose, it is necessary to verify the mutual independence of variables before landslide susceptibility mapping. Apart from that, it is crucial to remove the variables with close correlation by thoroughly analyzing the current situation in the study area. In this way, it is possible to avoid the inaccuracy and distortion in model assessment caused by the high correlation between variables [35]. Therefore, the correlation coefficient was used to assess the mutual independence between variables. To be specific, the values of less than 0.3 indicate that the factors are irrelevant to each other, the values ranging between 0.3 and 0.5 indicate low correlation, the values ranging from 0.5 to 0.8 indicate moderate correlation, and the values of no less than 0.8 indicate high correlation [36]. After verification (Figure 2a), there are high correlations between elevation (X10), undulation (X11), and other variables (X1…X9). The correlation coefficients reach as high as 0.53 and 0.89, both of which exceed 0.5. Besides, they are removed from the index system by taking into account reality. After elevation and undulation were eliminated, the correlation test was conducted again on the remaining variables (Figure 2b). It was discovered that all the correlation coefficients between the variables fell below 0.3, which suggests mutual independence. Therefore, the requirements of the model can be satisfied.

**Figure 3:**
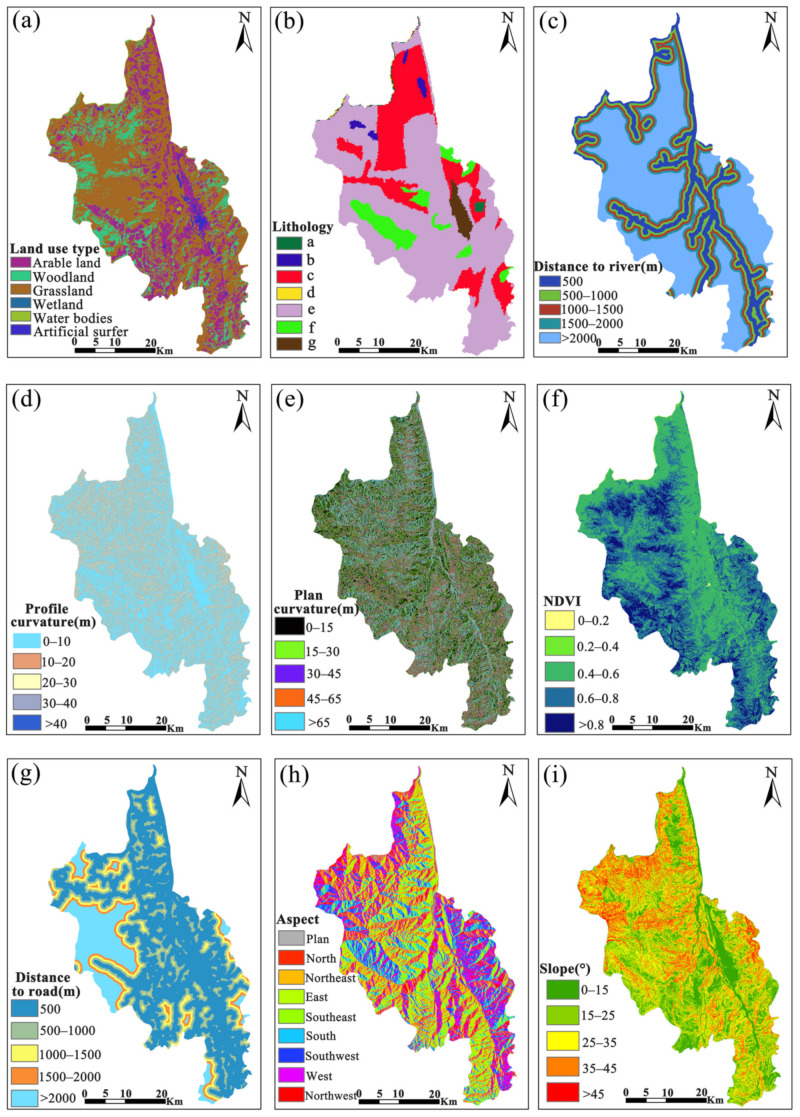
Classification chart of disaster-causing variables: (**a**) is the land use type, (**b**) is the lithology, (**c**) is the distance to a river, (**d**) is the profile curvature, (**e**) is the plan curvature, (**f**) is the NDVI, (**g**) is the distance to road, (**h**) is the aspect, and (**i**) is the slope. In (**b**), a represents acidic deep-formed rocks, b represents basaltic volcanic rocks, c represents carbonate sedimentary rocks, d represents neutral volcanic rocks, e represents mixed sedimentary rocks, f represents siliceous clastic sedimentary rocks, and g represents loose sediment.

In addition to the selection of LSM variables, the selection of mapping cells is another prerequisite for LSM modeling. In general, it involves raster cells, slope cells, and basin cells. Among them, the slope and raster cells are most used. However, the practical application of slope cells is constrained by the low operability, the difficulty in manipulation, the discontinuity of the obtained slope cells, and the heavy reliance on manual correction [37]. In contrast, raster cells demonstrate such advantages as regular shape, fast dissection, and high model computational efficiency, despite no favorable conditions created for characterizing topographic features. For this reason, the raster cell method was adopted in this paper for landslide susceptibility mapping. Besides, the 30 × 30 m cell was treated as the minimum mapping cell [38] so as to resample the final nine selected variables, unify the cell resolution size to 30 × 30 m, and grade each variable based on the experience of previous researchers and the pattern of hazard points distribution (Figure 3).

### 3.2. Landslide Susceptibility Models

#### 3.2.1. Logistic Regression Model (LR)

As one of the most used multivariate statistical methods, logistic regression (LR) is applicable to deal with complex nonlinear problems [39]. The advantage of LR is as follows. By introducing a suitable link function into the general linear regression, the variables may become either discrete or continuous, or any combination of both types. Besides, they do not necessarily conform to normal distributions. This method is effective in predicting the probability of occurrence or classifying a dependent variable when the dependent variable is a categorical variable, especially a binary dependent variable [40]. Among the models based on statistical learning, the logistic regression model performs better in reflecting the binary relationship between landslides and non-landslides, and in mapping the nonlinear function between landslide catalogues and their causative factors. It can be expressed as follows.

(1) $$\left\{\begin{array}{l} P(Y=1|X)=\frac{1}{1+e^{-z}}\\ Z=\beta_{0}+\beta_{1}X_{1}+\beta_{2}X_{2}+\cdot \cdot \cdot +\beta_{n}X_{n} \end{array}\right.$$

where, *P* represents the probability of landslide, the value range of which is [0, 1]; $X_{i}$ indicates the independent variable; *Z* denotes the relationship between the probability of landslide and each causative factor; and $\beta_{i}$ refers to the logistic regression coefficient of each causative factor.

The nine types of causal factors as selected in Section 3.1 were taken as independent variables, and the result of whether landslides occurred was treated as the dependent variable Y (1 for landslide occurrence and 0 for no landslide occurrence). With the attributed values of the variables assigned to each sample point, all sample values were inputted into SPSS26 for binary logistic regression analysis. In doing so, the probability value of landslide occurrence between 0 and 1 was predicted for each sample so as to generate the LSM in the Dongchuan district.

#### 3.2.2. Support Vector Machine Model (SVM)

As a nonlinear classification method, the support vector machine is premised on the principle of the Vapnik-Chervonenkis Dimension and structural risk minimization [41]. By means of nonlinear transformation, the input variables in the original space are mapped into a high-dimensional linear feature space. Then, the optimal decision function is constructed. The core idea is to make the data that are not separable in the original input space linearly separable by constructing an optimal separation hyperplane. The model performs better in solving such realistic problems as small samples, high dimensionality, and nonlinearity, which endows it with an excellent generalization capability [42].

Suppose $\left(x_{i},y_{i}\right)$ = 1,2, …, *n* for a linearly divisible sample, then the optimal hypersurface can be solved through the following function.

(2) $$\left\{\begin{array}{l} \min(\frac{1}{2}{\left\|\vec{w}\right\|}^{2}+C\sum_{i=1}^{n}\xi_{i}\\ y_{i}(\vec{w}\cdot {\vec{x}}_{i}+b)-1+\xi_{i}\geq 0\\ \xi_{i}\geq 0,i=1,2\cdot \cdot \cdot ,n \end{array}\right.$$

where *ω* represents the weight vector that determines the hyperplane direction, *b* indicatesthe deviation, $\xi_{i}$ denotes the relaxation factor, and *C* refers to the penalty factor. With the introduction of the Lagrange multiplier, the Wolf pair theory is applied to transform it into the following equivalent pair problem.

(3) $$\left\{\begin{array}{l} \max(\sum_{i}\alpha_{i}-\frac{1}{2}\sum_{i,j}\alpha_{i}\alpha_{j}y_{i}y_{j}({\vec{x}}_{i}\cdot {\vec{x}}_{j}))\\ \sum_{i}\alpha_{i}y_{j}=0,0\leq \alpha_{i}\leq C \end{array}\right.$$

Then, the resulting decision function can be used to classify the new sample data and it is expressed as follows.

(4) $$f(x)=\mathrm{sgn}(\sum_{i=1}^{n}y_{i}\alpha_{i}K(x_{i},y_{i})+b)$$

where $\alpha_{i}$ represents the Lagrangian multiplier, and $K(x_{i},y_{i})$ indicates the kernel function of the support vector machine. The performance of SVM models is closely related to the choice of kernel functions. Currently, the commonly used kernel functions include the linear kernel function, polynomial kernel function, radial basis kernel function, and Sigmoid kernel function.

In this paper, the widely used radial basis is chosen as the nonlinear mapping function for the model [43,44] which requires optimization for two types of parameters: the penalty factor *C* and the kernel function parameter Gamma. *C* represents a tunable constant that determines the degree of penalty for incorrect samples. Besides that, the larger *C* is, the heavier the penalty would be for errors. Comparatively, Gamma determines the distribution of the data after mapping to a new feature space, thus affecting the generalization performance. Besides, the larger Gamma is, the more likely it is for overfitting to occur, and vice versa [45]. To ensure the accuracy of the model, the grid search method [46] was adopted for hyperparameter tuning, which is aimed to arrange and combine the possible values of parameters *C* and Gamma (2^−10^ to 2^10^). In addition, all possible combination results were listed to generate a grid, thus determining the best parameter values. When the penalty parameter *C* was 0.5 and the Gamma parameter was 0.5, the highest accuracy reached was 87.06%. Therefore, *C* = 0.5 and Gamma = 0.5 were taken as the optimal parameters of the model. On this basis, the SVM model was constructed by adopting the framework of Libsvm [47] under Matlab language, so as to obtain the landslide probability value of each sample and to establish the LSM based on SVM in the Dongchuan district.

### 3.3. SBAS-InSAR

The SBAS-InSAR technique was first proposed by Italian scholars Berardino et al. [48] in 2002. It is intended mainly to decompose SAR images into several small baseline sets based on the spatial and temporal baseline thresholds. In the meantime, least squares are used to calculate each subset, so as to obtain surface deformation time series. This technique is effective not only in solving the decoherence and atmospheric effects caused by long spatial baselines but also in improving spatiotemporal coherence and increasing sampling frequency. The basic principle of it is as follows [49]:

Suppose there are *N* + 1 SAR images of the same area as captured at time *t_A_* to *t_B_*, and M differential interferograms generated for *N* + 1 SAR images, then it is satisfied that:

(5) $$\frac{N+1}{2}\leq M\leq \frac{N(N+1)}{2}$$

For the *j*th (*j* = 1, 2, …, *N* + 1) differential interferogram generated by the two images at the moments *t_A_* and *t_B_*(*t_A_* < *t_B_*), the interference phase at any point can be expressed as:

(6) $$\delta \varphi_{j}=\varphi_{tB}-\varphi_{tA}\approx \frac{4\pi }{\lambda }(d_{tB}-d_{tA})+\Delta \varphi_{j}^{topo}+\Delta \varphi_{j}^{atm}+\Delta \varphi_{j}^{noise}$$

With the removal of the atmospheric delayed phase, residual topographic phase, and noise phase, Equation (6) can be simplified into:

(7) $$\delta \varphi_{j}=\varphi_{tB}-\delta \varphi_{tA}\approx \frac{4\pi }{\lambda }(d_{tB}-d_{tA})$$

It is assumed that the deformation rate shows linearity at two adjacent time intervals so that the deformation phase of the *j*th interferogram can be written as:

(8) $$\delta \varphi_{j}=\sum_{k=tB,j+1}^{tA,j}\left(t_{k}-t_{k-1}\right)\cdot v_{k}$$

where *t* represents time, *δϕ* indicates phase, *λ* denotes the wavelength, *d* refers to the cumulative deformation, and *v* stands for the deformation rate.

InSAR technology has emerged in recent years for the early identification of geological hazards due to its robustness to various weather conditions, wide monitoring range, and high accuracy, which makes it widely applicable in landslide detection research [50,51]. However, the motion of slopes is three-dimensional in space. Due to the characteristics of sideview imaging performed by SAR sensors, the surface deformation as detected by InSAR technology is confined to the surface deformation occurring along the line of sight (LOS) direction of the satellite. In order to associate it with real-world deformation, many scholars have attempted to transform the deformation along the LOS direction into the deformation along the slope direction [52,53]. However, such a practice was not used in this study because slope-oriented deformation can play its role only in the case of translational landslides [54]. Through the field survey, it was discovered that most of the landslides that occurred in the study area involved the deformation caused in the vertical direction. Therefore, the experiments were conducted by using the ascending and descending orbits sentinel-1A data. Moreover, by taking into account different geometric parameters (Table 1), the deformation in the LOS direction was transformed into that along the vertical direction, so as to obtain the deformation information closer to the slope motion in the study area. The equation of its projection conversion is expressed as [55]:

(9) $$d_{Los}=d_{Nth}\sin\alpha \sin\theta -d_{Est}\cos\alpha \sin\theta +d_{Up}\cos\theta$$

In the Equation (9), there are three unknown variables (*d_Nth_, d_Est_, d_Up_*). For this reason, it is necessary to establish at least three equations for solving the equation. The geometric relationship of the near-polar flight of the SAR satellite makes it insensitive to the deformation occurring in the north-south direction. Therefore, when there are only two different SAR data sets that can be obtained, the components in the north-south direction can be ignored [56], so as to reduce the number of unknown variables. Then, Equation (9) can be simplified into:

(10) $$\left\{\begin{array}{l} d_{A_{Los}}=d_{Est}\cos\alpha_{A}\sin\theta_{A}+d_{Up}\cos\theta_{A}\\ d_{D_{Los}}=d_{Est}\cos\alpha_{D}\sin\theta_{D}+d_{Up}\cos\theta_{D} \end{array}\right.$$

where $d_{A_{Los}}$; $d_{D_{Los}}$; $d_{Est}$; $d_{Nth}$; $d_{Up}$ represent the projection components in the ascending and descending orbital LOS direction, east-west direction, north-south direction, and vertical upward direction, respectively, while *α* is the azimuth angle of the satellite flight direction, and *θ* is the incidence angle.

### 3.4. Integration

The purpose of integration is to improve the accuracy of LSM and minimize the misclassification results through SBAS-InSAR. In doing so, it is achievable to obtain more accurate LSM results for those landslide-prone areas. To achieve this goal, the LSM and SBAS-InSAR acquired vertical deformation rate Vup values as obtained from the model separately were reclassified in this study. Meanwhile, a value was assigned to each category: very low susceptibility = 1; low susceptibility = 2; medium susceptibility = 3; high susceptibility = 4; and very high susceptibility = 5. The SBAS-InSAR vertical deformation rate classification was determined by the standard deviation (σ = 8 mm/year): very low deformation (0–−8 mm/year) = 1; low deformation (−8–−16 mm/year) = 2; medium deformation (−16–−24 mm/year) = 3; high deformation (−24–−32 mm/year) = 4; and very high deformation (<−32 mm/year) = 5. Notably, since the deformation triggered by landslides is often manifested as subsidence, it is necessary to exclude the positive part of Vup if it exceeds 0, with positive values representing uplift and negative values representing subsidence. Then, depending on the Vup value, the susceptibility class of the LSM classification results increased. More specifically, *a* denotes the susceptibility class value as obtained by the model, *b* denotes the Vup class value, and *C* denotes the corrected value. It can be expressed as the following equation:

(11) $$C=\left\{\begin{array}{l} a=(b-a),a<b\\ a,\;\;\;\;\;\;a\geq b \end{array}\right.$$

According to Equation (11), the contingency matrix (Table 2) was constructed to integrate SBAS-InSAR and LSM. In this way, the cell susceptibility classes of those characterized by surface deformation can be updated, thus reducing the classification errors. When the value of the model-based landslide susceptibility class falls below that of the Vup class, the landslide susceptibility class increases by the difference between the two classification results. When the value of the model-based landslide susceptibility class equals or exceeds that of the Vup class, the original landslide susceptibility class remains unchanged. As shown in Table 2, for the area with a susceptibility class of 1, the updated value can be 0, +1, +2, +3, or +4 as the Vup value increases, and for the area with class 2, the updated value can be 0, +1, +2, +3, and so on.

## 4. Results

### 4.1. The LSM of the Dongchuan District

**Figure 4:**
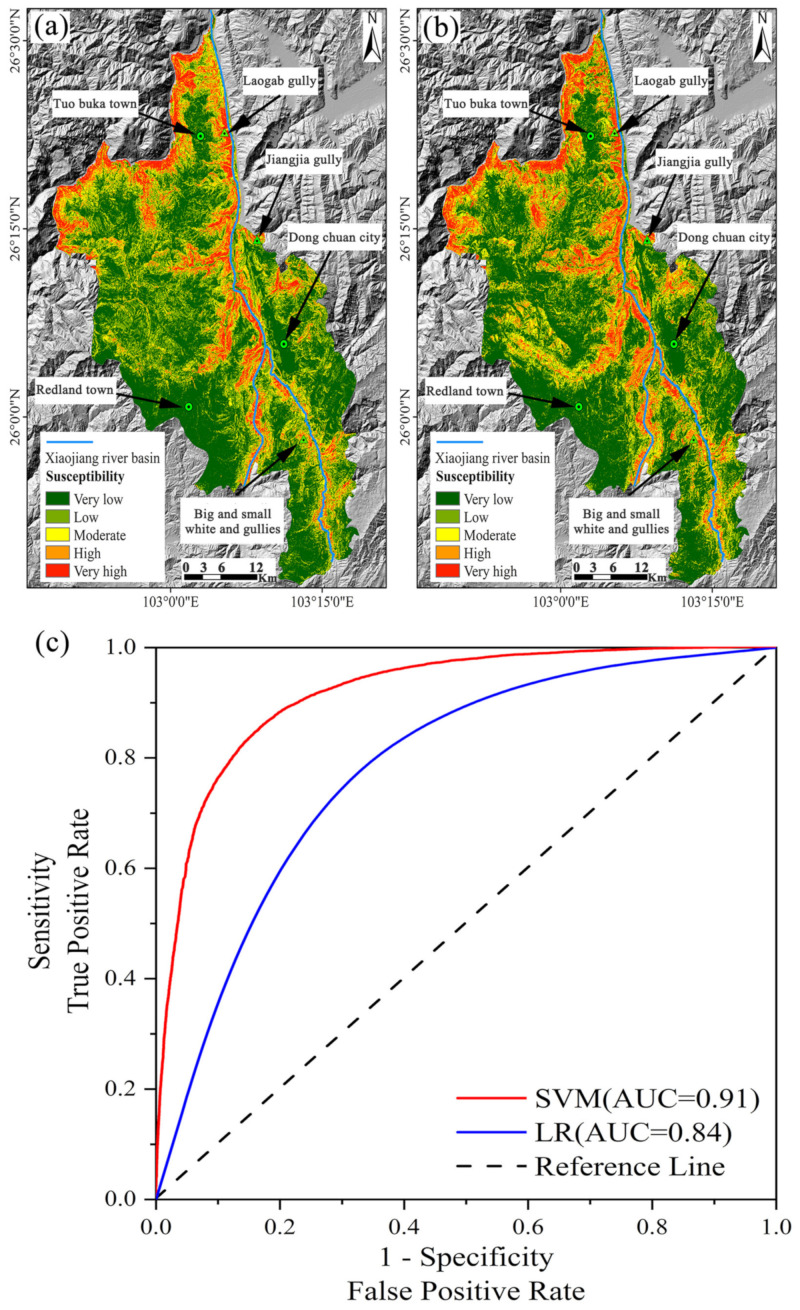
Landslide susceptibility map and ROC curve in the Dongchuan district. (**a**) is the landslide susceptibility map generated by the logistic regression model, (**b**) is the landslide susceptibility map generated by the support vector machine model, and (**c**) is the ROC curve.

The LR and SVM models as constructed in Part 3.1 were implemented by SPSS26 and Matlab2020b software. Then, the landslide probability values of each cell (30 × 30 m) in the study area were obtained and classified into five classes by using the natural breaks method [22,57] in the GIS environment. In ascending order, they are very low susceptibility, low susceptibility, medium susceptibility, high susceptibility, and very high susceptibility, respectively (Figure 4). On the whole, the two models maintain good consistency in the spatial distribution in those very low and very high susceptibility areas, especially the high susceptibility areas concentrated in the high mountain valley areas on both sides of the Xiaojiang River basin. This is attributable to the complexity of topography and geomorphology in the watershed, and to the long-term damage caused to the ecological environment. Ultimately, it led to the loss of ecological functions in most areas. Therefore, the units in the watershed were assigned a higher landslide susceptibility class. Apart from this, most of the remaining areas showed either very low or low susceptibility.

In order to validate LSM, both qualitative and quantitative perspectives were taken in this paper. From the qualitative perspective, three typical landslide areas and three stable areas, as illustrated in Figure 4a,b, were selected to test the LSM through their spatial correlation. Among them, Lao Gan gully, Jiang Jia gully, and Big and small-White mud gullies are located in the severely eroded mid-alpine geomorphic area. They feature not only relatively significant height differences in the terrain but also the deep cutting and accumulation of loose solids in the gully bed, which makes them the three major mudslide gullies most frequently struck by geological disasters in the Dongchuan District. From Figure 4a,b, it can be seen that the units within the three areas in the LSM are assigned an extremely high value of susceptibility, indicating the extremely high likelihood of landslides occurring in these areas relative to other areas. This is consistent with reality. The remaining three areas include the town of Tuobuka, the town of Redland, and the urban area of Dongchuan. As the densely populated residential areas in the Dongchuan District, they are characterized by a large number of artificial buildings, slight topographic undulations, and flat terrain. This leads to high stability and prevents the occurrence of landslides. According to the LSMs, as obtained from the two models, all three urban areas are located in the zone with very low susceptibility. Through qualitative verification, a preliminary conclusion can be drawn that the LSM as obtained by using the logistic regression model and support vector machine model is reasonable and macroscopically consistent with the actual performance. Quantitatively, it was achieved mainly through the ROC curve which provides an effective solution to assessing the validity of the classification. Besides, a higher value of the area under the curve (AUC) means a higher accuracy of prediction for the model [58]. As shown in Figure 4c, the AUC values are 0.84 and 0.91 for the logistic regression model and the support vector machine model, respectively. That is to say, the support vector machine model outperforms the logistic regression model in predicting landslide susceptibility in the Dongchuan district. Therefore, it is more suitable for obtaining the LSM in the area. However, its value is lower than 1, which suggests that there remain some classification errors in the LSM. It is thus necessary to correct them through appropriate methods, which is the focus of Section 4.3.

### 4.2. SBAS-InSAR

**Figure 5:**
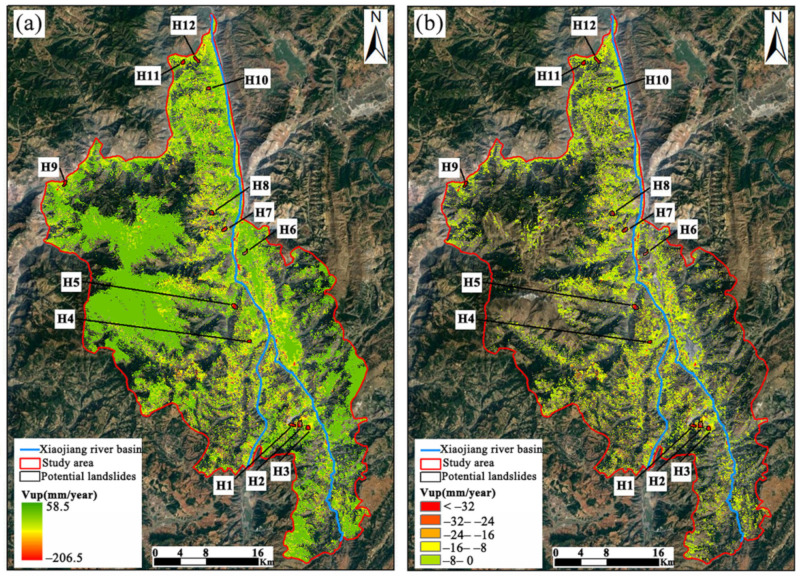
Vertical deformation rate results. (**a**) shows the results of the vertical deformation rate in the complete Dongchuan district, and (**b**) shows the results of the vertical deformation rate after excluding Vup > 0 and the flat area.

With 0.7 as the coherent threshold, the line of sight (LOS) deformation velocity (V_Los_) was calculated in SBAS-InSAR processing. Besides this, through the raster calculator in Arcgis 10.2, the results of the deformation rate in the vertical direction of the study area were obtained by projecting them to the vertical direction in accordance with Equation (10) (Figure 5a). In addition, the vertical deformation rate results must be resampled to maintain the same cell size (30 × 30 m) as LSM. Since this experiment focused only on landslides and the surface deformation in flat areas was ignored, the areas with a slope of less than 5° and Vup values higher than 0 were excluded. Therefore, only the subsidence results in sloped areas were retained (Figure 5b). In general, landslide surface deformation is part of the essential information required for analyzing the stability and hazard of landslides and for warning of collapse [59]. From Figure 5, it can be seen that the areas with significant changes to vertical deformation in the Dongchuan District concentrate on both sides of the Xiaojiang River basin, conforming to an “I” distribution. In comparison, the areas with lower deformation rates are distributed throughout the densely populated urban areas, which is consistent with the division of very high and very low susceptibility areas in LSM. According to the InSAR results, most of the areas exhibited deformation but to different degrees, with the maximum vertical deformation rate reaching as high as −206.5 mm/year. There were 12 potential landslide areas identified with obvious subsidence centers and denoted as H1, H2…H12, respectively. Among them, nearly all the cells in the seven hidden areas fall within the high and very high susceptibility zones according to the landslide susceptibility results. However, over half of the cells in the remaining five hidden areas concentrate in the very low and low susceptibility zones, which is inconsistent with the vertical deformation rate performance. It is indicated that there may be a classification error in some areas for the original LSM, as confirmed by the AUC value of 0.91 in Section 4.1.

### 4.3. Integration

**Figure 6:**
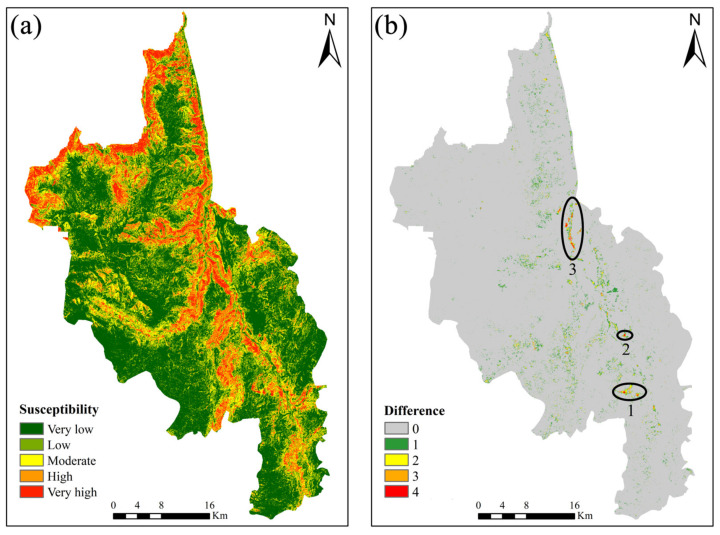
LSM and difference graph after correction. (**a**) shows the LSM after the integrated SBAS-InSAR correction, and (**b**) shows the difference graph before and after the correction. (0, 1, 2, 3, 4 represent the correction class value respectively).

As revealed by the evaluation results of the two models in Section 4.1, SVM (0.91) achieves a higher accuracy than LR (0.84), which makes it more suitable for the generation of LSM in the Dongchuan district. Therefore, the LSM was selected under the optimal model SVM. Besides this, the 30 × 30 m grid was constructed by Arcgis 10.2 to traverse the LSM and the reclassification class values of each cell in the vertical deformation rate results. Then, the integration with SBAS-InSAR was implemented by using Equation (11) and the contingency matrix (Table 2), so as to generate the new LSM (Figure 6a). The new LSM is characterized by the shrinkage of very low and low susceptibility areas and by the expansion of very high and high susceptibility areas. Compared with the LSM generated by the original SVM, the percentage change of each class cell in the new LSM is less significant. That is to say, most of the study area can be correctly classed by the original LSM, despite a small minority of the area requiring correction by SBAS-InSAR. To intuitively assess the magnitude of change between the original LSM and the new LSM, the differences between them were calculated by using the susceptibility class values of each unit (Figure 6b). These differences were analyzed quantitatively, as shown in Table 3. According to the new LSM, the susceptibility class of unit 66,094 increased (59.48 km^2^): unit 49,528 (44.57 km^2^) increased by 1 class, unit 12,061 (10.85 km^2^) increased by 2 classes, and unit 4478 (4.06 km^2^) increased by 3 classes or more. It is demonstrated that after the SBAS-InSAR technology was applied, the susceptibility of 66,094 classification error units in the original LSM had been corrected. What is noteworthy are the regions with an increase by 3 classes and more. This is because a sharper increase indicates a lower susceptibility in the original LSM and more significant classification errors.

In Figure 6b, there are three typical classification error regions (numbered 1–3) circled with significant changes in susceptibility class, which is purposed to better explain the variations and to verify the outcomes of correction. According to the field survey and remote sensing images, area 1 was found to be extremely hazardous. Around the two landslide potentials (H2 and H3) in area 1, there are a large number of villagers taking residence and engaging in production activities. If this area is classified as stable for land use that should be banned, it will cause severe loss of life and properties in the case of a landslide without any precautionary measures in place. Compared with areas 2 and 3, this area deserves more attention. Therefore, in the following section, our focus is on the two slopes shown in the typical area 1.

### 4.4. Results of Specific Case

In this section, our focus is to analyze two slopes in the typical area 1: H2 and H3. This is achieved through a combination of three means: remote sensing images, InSAR monitoring results, and field visits.

**Figure 7:**
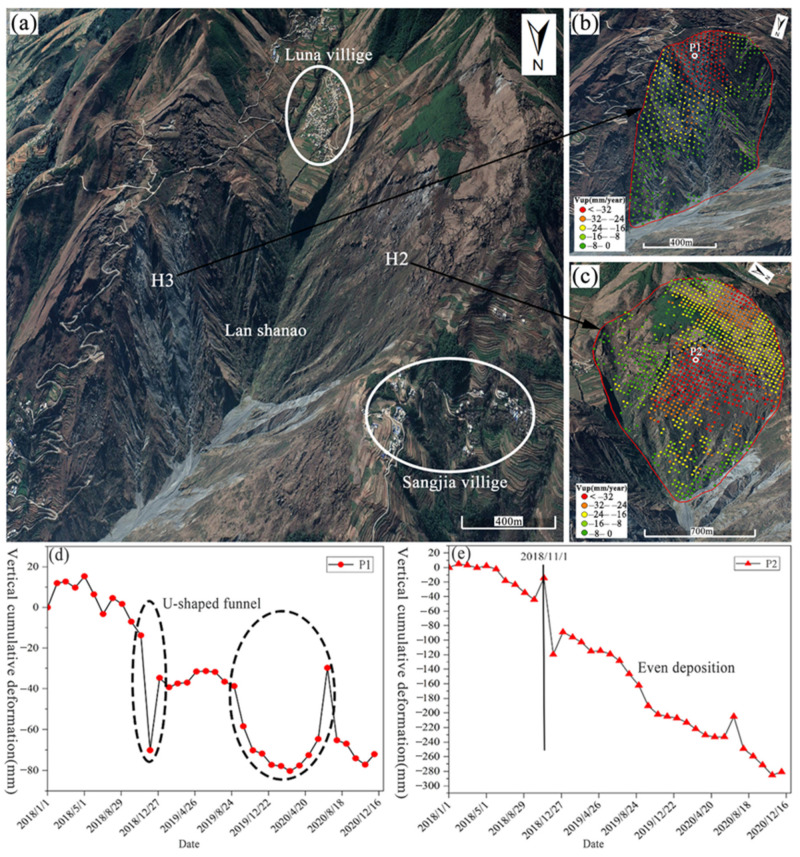
Typical regional remote sensing images and cumulative deformation time series of feature points. (**a**) is remote sensing image, (**b**,**c**) are H3 and H2 regional zooms, respectively, and (**d**,**e**) are the cumulative deformation time series of feature points P1 and P2, respectively.

H2 is located on the westward slope of the rotten mountain depression (Figure 7a). The slope in this area ranges mostly between 25–30°. It is steep at the top and gentle at the bottom, resembling a lap chair, which makes it more likely for landslides to occur. Mixed sedimentary rocks are the main material that constitutes this slope, which results from the extrusion of the Xiaojiang Fracture Zone, with broken and loose rock masses, weak shear strength, and weathering resistance. Therefore, it is highly susceptible to the effects caused by wind and rainfall. Especially in the middle and back edge, the signs of deformation are evident. Besides, some open structural surfaces facilitate the infiltration of rainwater into the slope. This leads to the erosion of geotechnical soil and increases the geotechnical capacity, thus accelerating the deformation of the slope. In addition, two villages, Luna village and Sanjia village, are located on both sides of the slope, with plenty of agricultural land surrounding them. Due to engineering construction and irrigation, the deformation caused to the slope is accelerated to different degrees. As suggested by the SBAS-InSAR monitoring results (Figure 7c), the vertical deformation rate in this area exceeds −32 mm per year, which indicates the ongoing deformation of the slope. Judging from the trend of cumulative deformation (Figure 7e) over time, the overall deformation of the slope is significant, and the cumulative deformation reaches as high as 280 mm over three years. In November 2018, the change was accelerated significantly, with the cumulative deformation reaching as high as 120 mm in just over a month. Initially, it was speculated that a minor landslide occurred during this period.

H3 is located on the east-facing slope of the Ragged Mountain Depression (Figure 7a). Across this area, the slope exceeds 35° in most cases, with carbonate sedimentary rocks as the dominant components. The slope plane resembles a long tongue on the whole, featuring a local stepped slope. The vegetation in the area is sparsely distributed, the bedrock structure is destroyed by tectonic movement, the development of internal pores and fissures is extraordinary, and the cracks tension and shear can be found all over the slope body. Recharged by rainfall and hillslope runoff, stagnant layers tend to develop in the interbedded parts of fine-grained soils or strongly weathered bedrock surfaces, where the softening and flow of water can prompt the loose accumulation to slide as a result of gravity [27]. As can be seen in Figure 7b, the maximum sliding of the slope occurs at the broken bedrock face in the back edge part, which exceeds −32 mm/year. Besides this, the deformation rate gradually decreases as the slope is reduced. By taking into account the trend of cumulative deformation in this area (Figure 7d), it can be clearly seen that the slope body experienced severe deformation twice between November 2018 and June 2020. The deformation increased first and then decreased, thus forming two large “U” shaped sinkholes. Especially, the first settling funnel indicates a brief but significant deformation. Before the formation of the second funnel, there is a level-off period, during which there is almost no deformation occurring to the slope. Different from the first subsidence funnel, the second one becomes relatively gentle, lasting nearly one year. It is indicated that after a level-off period, the deformation of the slope resumes, and it maintains a slow pace thereafter. The slope presents a major safety risk to the road leading to Luna village. Once the slope destabilizes and triggers a landslide, it will destroy the road and cut off the connection between the village and the outside world.

**Figure 8:**
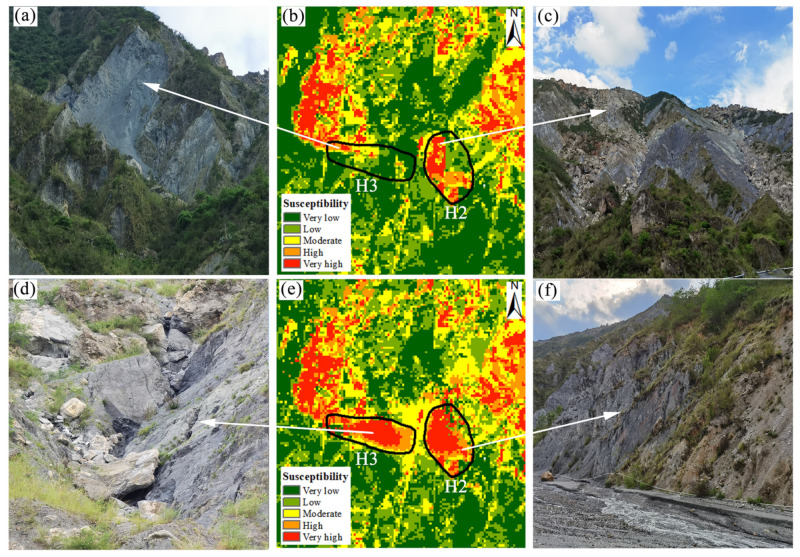
Fieldwork and susceptibility maps before and after correction of typical areas. (**c**,**f**) are field maps of the H2 region, (**a**,**d**) are field maps of the H3 region, (**b**) is the pre-correction susceptibility map, and (**e**) is the post-correction susceptibility map.

In addition, these two areas were further studied through field investigation. It was revealed that the rock and soil bodies in the two landslide-prone areas of H2 and H3 are broken and loose, vertical fissures are in development, and such loose accumulations as fallen rocks and crumbling occur commonly due to sliding and water flow, and there are plenty of minor, localized slides, crumbling, and flowing in development (Figure 8a,c,d,f). The results of field exploration are well consistent with the deformation texture characteristics of remote sensing images and the trend of InSAR cumulative deformation variables, which confirms a greater possibility that these two slopes could develop into landslides. However, it is worth noting that for the area with such evident landslide characteristics, most of the cells in the LSM as obtained by SVM show either very low or low susceptibility (Figure 8b). That is to say, in the original LSM, the two slopes stabilize, and the possibility of landslides is extremely low. Obviously, the susceptibility class evaluated using the SVM model underestimates the real susceptibility of the area.

According to the LSM, as obtained by integrating SBAS-InSAR, the cells within the slope area are integrated into the dynamic surface deformation information, which improves the susceptibility class significantly. Besides this, the original low and very low susceptibilities are corrected into high and very high susceptibilities (Figure 8b). Whether it is from remote sensing images, InSAR deformation rate results, or field survey results, it can be seen that the corrected susceptibility class is more similar to the landslide characteristics exhibited by the actual slope, which proves the reliability of the corrected results and highlights that the landslide prediction accuracy of the integrated SBAS-InSAR generated LSM is better than that of the original LSM.

## 5. Discussions

A comparison between the original and corrected LSM for two specific areas (H2, H3) revealed that the proposed method in this paper is capable of correcting the susceptibility class of classification error cells and producing a more reliable LSM. In detail, in the InSAR deformation results, two typical areas (H2, H3) showed obvious subsidence trends, and the deformation rate of the slope surface exceeded −32 mm/year (Figure 7b,c), and the deformation signs of the leading and bake edge were significant, with the maximum cumulative deformation reached 70 mm and 280 mm, respectively (Figure 7d,e). In the images, rockfalls, and collapse development, the rock was severely weathered and broken. The possibility of developing into a landslide under favorable conditions is extremely high, which is consistent with the corrected susceptibility class, i.e., high landslide probability corresponds to the high susceptibility class. In addition, through a comparison performed only by others with the LSMs obtained from the Dongchuan district based on the model, it was found that in the low susceptibility areas, the LSMs obtained by using the method proposed in this paper are basically consistent with those obtained by previous studies. Besides this, the low susceptibility classes are assigned to the town areas such as the flat terrain of the Redland and the Tuobuka. As for the high susceptibility areas, this paper integrates the SBAS-InSAR technique to improve the susceptibility class of classification error cells. Therefore, different results from previous studies are displayed. For example, in the LSM of the Dongchuan district obtained using RF [29], some high mountain areas on both sides of the Xiaojiang River basin with severe soil and water loss and large surface deformation rate (Figure 6b area 3) were assigned a medium susceptibility class; in the LSM of Dongchuan district obtained based on the combination assignment method [31], the big and small white mud gullies with frequent geological hazards were assigned a low susceptibility class. On the contrary, in the LSM obtained by the method proposed in this paper, these areas belong to very high susceptibility areas, and the susceptibility class is closer to that of the actual slope surface rock fragmentation and tension fracture dense state.

Nevertheless, the method shows some shortcomings because not all misclassifications in the region can be corrected, which depends on the integrity of the InSAR data results. In other words, the classification errors can be corrected only when valid In-SAR deformation values can be obtained in the region. As shown in Figure 5a, there are many null regions in the deformation rate maps as obtained by the SBAS-InSAR technique. This results primarily from the limitations of the Sentinel-1A sensor itself. In those areas with high vegetation cover, the C-band wavelength (~5.6 cm) is not capable of penetration, thus leading to the out-of-coherence phenomena that arise when there are null values in the deformation rate map. In these regions, the lack of InSAR data results can make it difficult to assess the susceptibility class in a reasonable way. To address this problem, L-band SAR sensors can be used due to their wavelength of 30–15 cm, which improves the penetration of the radar. As a result, the temporal decoherence effect caused by vegetation cover is reduced, which makes it more suitable for applications in those areas with dense vegetation [60]. However, because L-band SAR data are expensive to collect and require a longer revisit period relative to C-band Sentinel-1A data, it is less sensitive to surface deformation. Therefore, it was discounted from this experiment.

In addition to applying SBAS-InSAR technology, the other methods used to reduce misclassification may include manual inspection, light laser detection and ranging (LiDAR), and unmanned aerial vehicle (UAV) photogrammetry. However, since most of the Dongchuan district is covered by a high mountain valley, the traditional manual one-by-one ranking method is rendered ineffective. Although in different ways, LiDAR and UAV photogrammetry can be relied on to identify a single landslide in a highly accurate way. In terms of wide-area landslide discrimination, however, there are such disadvantages as it being a lengthy operation, high labor and financial costs, and the difficulty in popularization. In light of this, it is believed that the weakness of the InSAR technique in the loss of coherence for those areas with dense vegetation cover is acceptable. This is because, in densely vegetated areas, landslides are often less likely to occur due to the soil consolidation and slope protection of vegetation rhizomes. According to the original LSM (Figure 6), the areas with dense vegetation cover concentrate in the very low and low susceptibility zones, and their susceptibility classification is consistent with reality. It is indicated that the probability of being misclassified is extremely low. From a practical perspective, it is believed as a relatively feasible solution to correct classification errors and improve the reliability of LSM by applying InSAR technology. Especially, the free access to Sentinel-1A data makes this method widely applicable.

## 6. Conclusions

In order to address the classification error in LSM, this paper proposes to reduce the classification error and improve the reliability of LSM by constructing the contingency matrix, which achieves the integration of SBAS-InSAR and LSM. In the experiment, LR and SVM were used to generate an LSM for the Dongchuan district with a 30 × 30 m cell resolution, respectively, while the model was evaluated for accuracy by using an ROC curve. Besides this, the LSM under the optimal model SVM was adopted for integration with SBAS-InSAR, so as to correct the classification errors in the original LSM. By combining remote sensing images, InSAR results, and fieldwork, the typical areas with significant changes before and after correction were demonstrated and analyzed. On this basis, the following conclusions were drawn:

(1) The LSM as obtained from LR and SVM models was evaluated from both qualitative and quantitative perspectives. Qualitatively, the spatial distribution of the very high and very low susceptibility areas is consistent with reality. Besides this, those very high susceptibility areas are concentrated in the geological hazard-prone areas on both sides of the Xiaojiang River basin, while the very low susceptibility areas concentrated in the urban areas with flat topography. Quantitatively, the AUC values under the two models are 0.84 and 0.91, indicating that the SVM model is more accurate than the LR model under the same conditions. Therefore, it is more suitable for obtaining an LSM in the Dongchuan district.
(2) During the period from January 2018 to January 2021, the vertical deformation rates in the study area ranged from −206.5 to 58.5 mm/year. There were 12 landslide potential areas identified with obvious subsidence centers, of which 7 potential areas matched well with the landslide susceptibility class. However, there were 5 potential areas assigned a lower susceptibility class, which contradicted the reality. It is indicated that some of the areas were misclassified in the original LSM, which required improvement.
(3) Compared with the original LSM, the susceptibility class of 66,094 classification error cells (59.48 km^2^) was corrected in the LSM obtained by integrating the SBAS-InSAR technique. The areas with large differences in class variation showed obvious landslide characteristics in remote sensing images, InSAR deformation results, and field inspection, which is closer to the corrected susceptibility class. It is suggested that the method is effective in correcting the classification errors, which further enhances the reliability of the LSM.

In summary, this study is essential for improving the reliability of LSM in detecting classification errors and correcting them on a large scale. It can be applied to assist the planning and decision-making departments in correcting the classification errors in LSM fast and efficiently, thus providing more support for decision-making on regional disaster prevention and mitigation as well as land use planning.

## Figures and Tables

**Table 1:** Sentinel data parameters used in this study.

	Ascending Orbit	Descending Orbit
Track	128	62
Wave	C	C
Polarization mode	VV polarization	VV polarization
Average angle of incidence (°)	41.98	36.93
Average azimuth (°)	−12.43	−167.53
Time span	7 January 2018–3 January 2021	9 January 2018–5 January 2021

**Table 2:** Contingency matrix applied to the LSM considering the average Vup in each cell. (The susceptibility degree from 1 to 5 represents very low, low, moderate, high, and very high, respectively).

	V_up_(mm/year)					
		0–−8	−8–−16	−16–−24	−24–−32	<−32
	1	0	+1	+2	+3	+4
Susceptibility degree	2	0	0	+1	+2	+3
	3	0	0	0	+1	+2
	4	0	0	0	0	+1
	5	0	0	0	0	0

**Table 3:** Comparison of the original landslide susceptibility assessment degrees with the revised degrees after correction.

Susceptibility Degree	Original LSM		New LSM		Susceptibility Degree Increase	
Class	NO. Cells	%	NO. Cells	%	Class	NO. Cells
1	986,793	47.78	930,191	45.03	0	1,999,531
2	358,563	17.35	394,492	19.10	+1	49,528
3	316,316	15.31	329,650	15.96	+2	12,061
4	207,117	10.03	210,570	10.19	+3	3174
5	196,836	9.53	200,721	9.72	+4	1331

## Data Availability

The data are not publicly available as they involve the subsequent application of other studies.
